# Environmental Biotechnology: Current Advances, New Knowledge Gaps, and Emerging Issues

**DOI:** 10.1155/2015/814529

**Published:** 2015-12-14

**Authors:** Abd El-Latif Hesham, T. Komang Ralebitso-Senior, Yu Zhang, Qing X. Li

**Affiliations:** ^1^Genetics Department, Faculty of Agriculture, Assiut University, Assiut 71526, Egypt; ^2^School of Science and Engineering, Teesside University, Borough Road, Middlesbrough, Tees Valley TS1 3BA, UK; ^3^Research Centre for Eco-Environmental Sciences, Chinese Academy of Sciences, P.O. Box 2871, Beijing 100085, China; ^4^Department of Molecular Biosciences and Bioengineering, University of Hawaii at Manoa, Honolulu, HI 96822, USA

The term “environmental biotechnology” encapsulates a wide and dynamic spectrum of topics including bioremediation, biofiltration, wastewater treatment, biodegradation, waste management, and biofuel production. All these disciplines are underpinned by complex interacting microbial communities; hence exciting research has been undertaken and increasingly driven by cutting-edge ecogenomic techniques. However, understanding of microbial diversity, population, and structure for sustainable environmental management remains largely a “black box.” Thus exciting findings identified new knowledge gaps and, inevitably, engendered novel research questions with inventive methodologies.

This special issue is focused on original research and review articles that (i) present a critical and succinct appreciation of the environmental biotechnology state of the art, (ii) identify emerging areas of research, (iii) illustrate the applications of innovative molecular techniques, and (iv) suggest updates of established protocols.

P. Munguia-Fragozo and his colleagues have systematically reviewed the omic technologies for microbial community analysis. They discuss the potential applications of current “omics” such as denaturing gradient gel electrophoresis, microscopy using fluorescence* in situ* hybridization, and/or cloning rRNA gene fragments and bioinformatic tools to characterize the microbial community of aquaponic systems. Also they mentioned that metatranscriptomics, proteomics, and metabolomics can provide information of functional analyses in microbial communities at different levels of gene expression, protein translation, and, more recently, the metabolite network, respectively. They indicated essential roles of “omic” approaches such as metagenomics and metatranscriptomics on future studies of microbial diversity in aquaponic biosystems.

C. Varrone et al. reported their research on the selection and adaptation of mixed microbial cultures (MMCs), able to ferment crude glycerol generated from animal fat-based biodiesel and produce building-blocks and green chemicals. Various adaptation strategies have been investigated for the enrichment of suitable and stable MMCs, trying to overcome inhibition problems and enhance substrate degradation efficiency, as well as production of soluble fermentation products. Repeated transfers in small batches and fed-batch conditions were applied. They demonstrated that next generation sequencing represented a useful tool to monitor the changes in microbial composition of MMCs, highlighting the development of a glycerol consuming community (with numerous strains belonging to the genera* Clostridium*,* Klebsiella*, and* Escherichia*), thus confirming the effectiveness of the enrichment strategy.

L. Kiseleva et al. have focused their work on the isolation and partial characterization of an electrogenic bacterium* Thalassospira* sp. strain HJ from a magnetic particle-enriched portion of a marine tidal sediment. They described the first report of electrogenic behavior within the genus* Thalassospira*. They recommended that a more extensive study would be needed to determine whether the proportion of electrogenic bacteria obtained from this magnetic particle enrichment procedure exceeds that found in the environment. With their diurnal patterns of flooding and diversity of mineral components, tidal sediments should be rich environments to bioprospect for electrogenic bacteria.

Development of an* in situ* microfluidic biosensor for the detection of phenol, based on laccase from* Trametes pubescens* with flow-injection and amperometry as the transducer method, is reported by J. C. Gonzalez-Rivera and J. F. Osma in their study of “Fabrication of an Amperometric Flow-Injection Microfluidic Biosensor Based on Laccase for* In Situ* Determination of Phenolic Compounds.” The microfluidic biosensor showed better analytic characteristics than previous biosensors, such as the lower limit of detection and increased sensitivity. Moreover, the optimum operational conditions of temperature, pH, injection flow rate, and redox potential were established. Thus the microfluidic device can be applied directly to* in situ* operation while its fabrication procedure can be introduced for industrial applications.

Lead biosorption by* Klebsiella* sp. 3S1 isolated from a wastewater treatment plant was investigated by A. J. Muñoz et al. through a Rotatable Central Composite Experimental Design. According to their results, the biosorption pathway can be described by a two-step process, one rapid, almost instantaneous, and another slower, both contributing significantly to the overall biosorption. The model that fits the experimental results best was pseudo-second order. The mechanism study revealed that lead ions were bioaccumulated into the cytoplasm and adsorbed on the cell surface. Also, the bacterium* Klebsiella* sp. 3S1 had a good potential in the bioremoval of lead in an inexpensive and effective process.

The objective of a study done by M. E. Silva et al. was to assess antagonism of metabolites produced by nematophagous fungi and their effectiveness on* Haemonchus contortus* infective larvae (L3). Their findings showed the existence of fungal antagonism on the production of reproductive structures between species with potential use for control of gastrointestinal nematodes of domestic animals. The authors recommended that biotic application of nematophagous fungi, specifically for environmental control of gastrointestinal nematodes, must be investigated thoroughly in order to evaluate success of the parasite control program before implementation* in situ*. Another study reported by M. E. da Silva et al. aimed at evaluating the predatory activity of* Duddingtonia flagrans* and* Clonostachys rosea* and their combined impacts on infective larvae (L3) of* H. contortus* in grass microplots that were maintained in a protected environment. Since the efficiency was 74.5%, it was suggested that the tested association (*Clonostachys* +* Duddingtonia*) could be explored further in future studies of biological control. According to the researchers, this was the first report of* C. rosea* and* D. flagrans* association for the control of* H. contortus* in environmental conditions.

The risks of contracting staphylococci food poisoning from the consumption of improperly manufactured salami and the possibility of these food matrices being reservoirs for antibiotic resistance were evaluated by R. S. C. Nunes et al. Nineteen distinct coagulase-negative staphylococci (CNS) strains were found in commercial and artisanal salami. The CNS strains were identified by sequencing of a 16S rDNA region and the phylogenic relationships between the enriched species were established. The presence of multiple genes encoding the classical and newly described* se/sel* and* tstH1* toxins in the CNS genomes was investigated. The risk of food poisoning was then assessed by evaluating the ability of the CNS strains in transcribing and expressing the classical and newly described enterotoxins* in vitro* by using real-time PCR and enzyme-linked immunosorbent assays. The resistance of the isolated strains to antimicrobial agents of therapeutic importance in staphylococci infections was also evaluated.

Finally, J. H. Kwon et al. described the optimization of planetary mill pretreatment and saccharification processes for improving biosugar production from* Pinus rigida* wood waste. They demonstrated that milling can be used to obtain high levels of glucose bioconversion from woody biomass for biorefinery purposes.

All of these papers show how biotechnology and molecular tools can have a significant benefit in a wide range of environmental biotechnology research. It is hoped that readers will relate to the highlighted knowledge gaps and use these to identify new or related ones. All these should, inevitably, engender novel research questions to be addressed subsequently with inventive methodologies.

